# ASSOCIATION OF CUMULATIVE RISK FACTORS AND HANDGRIP STRENGTH AMONG COMMUNITY-DWELLING OLDER ADULTS IN INDIA

**DOI:** 10.1093/geroni/igae098.1771

**Published:** 2024-12-31

**Authors:** Snehal Kulkarni

**Affiliations:** Public Health Foundation of India, Delhi, Maharashtra, India

## Abstract

**Objective:**

The present study aims to establish the reference values of HGS in Indian older adults and to evaluate the influence of cumulative risk factors on HGS.

**Methods:**

This cross-sectional study was conducted in the Pune city of India among 1016 community-dwelling older adults. Data on demographic characteristics, functionality, chronic illness, and physical activity was collected using a structured questionnaire. Objective measurements such as slowness of walking and balance impairment were measured using the Timed-up and go test and sharpened Romberg’s test. Descriptive statistics, independent t-test, and one-way ANCOVA were to examine the influence of risk factors on HGS.

**Results:**

The mean HGS of the study group was 16.28 + 5.84 kgs. Male older people had a higher handgrip (20.37±6.91 kg) than females (14.29±5.54 kg). For both men and women, handgrip strength decreased with increasing age. The results show that participants with 4 or more risk factors had significantly low handgrip strength (14.18 kg) than participants with no risk factors (19.62 kg). Functional decline, lack of exercise, and impaired balance also reduced the handgrip among the study participants.

**Conclusion:**

The values of handgrip strength reported in the current were lower than those reported in other studies. Accumulation of more than 4 risk factors drastically decreases the handgrip. Thus, evaluation of handgrip strength can be used as a feasible strategy to improve the health status of community-dwelling older adults.

